# Ulcerative tuberculosis

**DOI:** 10.1016/j.idcr.2021.e01312

**Published:** 2021-10-16

**Authors:** Jesús Rojas Jaimes

**Affiliations:** Facultad de Ciencias de la Salud, Universidad Privada del Norte, Lima, Peru

Tuberculosis (TB), one of the most devastating endemic diseases in the world, has been aggravated by the HIV-AIDS epidemic [1]. The incidence of cutaneous TB, a rare chronic manifestation (1% of all TB cases), has become more frequent not only because of the epidemic, but because of the increased use of pharmaceutical immunosuppressants. Cutaneous tuberculosis occurs in a variety of clinical forms that depend on how bacilli reach the skin and the immune status of the individual [2]. Cases at the cutaneous level are usually spread by blood and/or lymphatic pathways from a primary focus, although they may be introduced directly by trauma to the skin or mucous membranes in certain cases [3].

This case is a 23-year-old immunocompetent male patient with pulmonary tuberculosis diagnosed by microscopy two and a half months before the admission to the hospital where the case began to be studied, without cultivation or follow-up of treatment by the patient who was involved in illegal mining. The patient reported trauma due to a blow to the chest area one and a half months before the appearance of ulcers in the chest area.

Drugs used at the beginning of the hospitalization were isoniazid (5 mg/kg), rifampin (10 mg/kg), pyrazinamide (30 mg/kg), ethambutol (25 mg/kg) and streptomycin (20 mg/kg). The patient had anemia at the beginning of the hospitalization (hematocrit 22%) and received personalized anti-tuberculosis treatment due to the adverse effects observed in the patient 2 weeks after starting treatment in the hospital. The patient reported liver dysfunction and febrile processes. Therefore, two weeks later, individual challenges were performed with isoniazid (15 mg/kg), rifampin (10 mg/kg), pyrazinamide (50 mg/kg), ethambutol (50 mg/kg) and streptomycin (25 mg/kg). A new sputum sample was collected and sent for testing, which was positive for *Mycobacterium tuberculosis* MDR (rifampicin, isoniazid, and ethionamide). Then, a biopsy of the ulcer was also taken for culture and tested positive for *Mycobacterium tuberculosis* with resistance to rifampin, isoniazid, ethionamide, and pyrazinamide. The patient died of an adverse reaction that caused hepatic and renal failure related to abnormal amounts of aminotransferase (AST 135 U/L, NV 5–40 U/L; ALT 160 U/L,NV 7–56 U/L), alkaline phosphatase (464 U/L, NV 50–136 U/L) and acid uric serum (14.6 mg/dL, NV 2.4–7 mg/dL), additionally, the clinical evaluation revealed hepatomegaly. Besides having had a marked edema in the left arm and foot. Organ failure was reported two weeks after starting treatment.

The diagnosis of Buruli ulcer was ruled out after identifying the etiologic agent as *Mycobacterium tuberculosis*, classified as endogenous cutaneous TB with characteristics of orificial TB, in which the patient had an endogenous spread from an initial location, such as the lung, via the blood or lymphatic system [2], [4]. Since our patient, first, had pulmonary tuberculosis and cutaneous tuberculosis later, it is assumed that the spread of the bacteria and subsequent skin ulceration must have spread from the lung parenchyma to the skin, stimulated by trauma to the chest area due to motorcycle accident (Fig. 1). It is important to note that malnutrition and trauma led to an uncontrolled local inflammatory process that caused the development of ulcers [5], from which *M. tuberculosis* MDR was cultured, and that the patient had adverse effects related to anti-tuberculosis drugs.

**Fig. 1:**
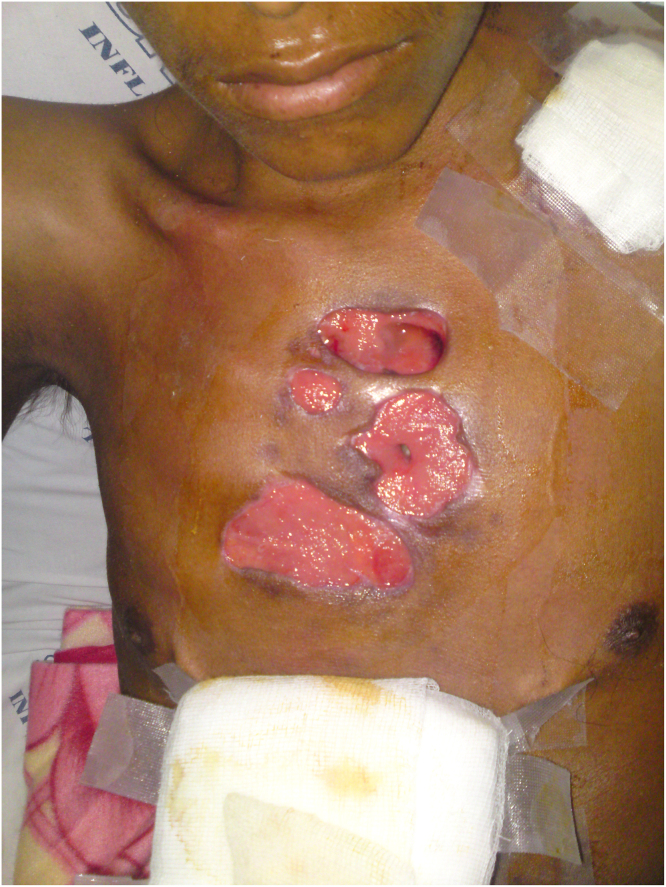
The image shows the ulcers. One of them reached the pleura.

## Funding

This research did not receive any specific grants from funding agencies in the public, commercial or non-profit sectors.

## Consent

In this case, no written informed consent was requested. There are no marks or identification features or patient identifiers on the images or accompanying text. Therefore, the intimacy of the patient is not violated.

## CRediT authorship contribution statement

JRJ looked after the patient, wrote and edited the manuscript. The author has read and approved the final manuscript.

## Declaration of competing interests

The author does not report conflict of interests.

## Author statement

Author Agreement Dear IDCases. I Jesús Rojas Jaimes I Agree with the policy that save the academic and ethics proposed by IDCases. Thank You.
